# Reaching substantive female representation among decision-makers: A qualitative research study of gender-related experiences from the health sector in Mozambique

**DOI:** 10.1371/journal.pone.0207225

**Published:** 2018-11-15

**Authors:** Talata Sawadogo-Lewis, Réka Maulide Cane, Rosemary Morgan, Mary Qiu, Amilcar Magaço, Kátia Ngale, Timothy Roberton

**Affiliations:** 1 Department of International Health, Johns Hopkins Bloomberg School of Public Health, Baltimore, Maryland, United States of America; 2 Health Systems Cluster, Instituto Nacional de Saúde, Ministry of Health, Maputo, Mozambique; Middlesex University, UNITED KINGDOM

## Abstract

**Background:**

Achieving significant female representation in government at decision-making levels has been identified as a key step towards achieving gender equality. In 2015, women held 39.6% of parliamentary seats in Mozambique, which is above the benchmark of 30% that has been suggested as the turning point for minority representation to move from token status to having a sizable impact. We undertook a study to identify gender-related barriers and facilitators to improving women-centered policies in the health sector.

**Methods:**

We conducted in-depth interviews with 39 individuals (32 women, 7 men) involved at a senior level in policy making or implementation of woman-centric policies within the Mozambique Ministry of Health and affiliated institutions. We used a semi-structured interview guide that included questions on difficulties and facilitating factors encountered in the policy making process, and the perceived role of gender in this process. We used both deductive and inductive analysis approaches, starting with a set of pre-identified themes and expanding this to include themes that emerged during coding.

**Results:**

Our data suggest two main findings: (1) the women who participated in our study generally do not report feeling discrimination in the workplace and (2) senior health sector perceive women to be more personally attuned to women-centric issues than men. Within our specific sample, we found little to suggest that gender discrimination is a problem professionally for female decision-makers in Mozambique. However, these findings should be contextualized using an intersectional lens with recognition of the important difference between descriptive versus substantive female representation, and whether “percentage of women” is truly the best metric for gaging commitment to gender equality at the policy making level.

**Conclusions:**

Mozambique’s longstanding significant representation of women may have led to creating an environment that leads to positive experiences for female decision-makers in the government. However, while the current level of female representation should be celebrated, it does not negate the need for continued focus on female representation in decision-making positions.

## Introduction and context

Gender equality is a priority on the global development agenda, as exemplified by the fifth Sustainable Development Goal (SDG): “Achieve gender equality and empower all women and girls”. Beyond its importance for human dignity and respect for human rights, empowering women leads to better health, education and economic outcomes [1]. SDG target 5.5 –“Ensure women’s full and effective participation and equal opportunities for leadership at all levels of decision-making in political, economic and public life”–explicitly points to female representation within different levels of decision-making as a factor in achieving gender equality [2].

There is evidence that increasing women’s representation in leadership positions leads to more equitable health outcomes, particularly for women and children [3]. Randomized trials suggest that “women in leadership positions in governmental organizations implement different policies than men and that these policies are more supportive of women and children” [4]. A field study in India showed that women policy makers tend to invest in interventions and infrastructure more closely linked with women’s concerns, such as clean drinking water, while male policy makers tended to invest more in infrastructure more closely aligned to men’s concerns and activities, such as irrigation systems for farming [3, 4]. Another study in India, by Bhalotra and Clots-Figueras (2014), suggested a link between increased political representation for women and decreased neonatal mortality. Increased female representation was found to have a positive association with women “attending antenatal care, taking iron supplements during pregnancy, giving birth in a government facility as opposed to at home […], and early initiation of breastfeeding” [5]. More generally, having a more balanced gender distribution in government—as well as in all organizations—might be beneficial since studies have suggested that women behave more honestly than men [6], which might be welcome change in some political arenas.

In this paper, “woman-centered policies” refer to policies which promote the health and wellbeing of biologically female individuals by addressing concerns which disproportionally affect the female sex (e.g. maternal health and child health focus areas). We also use “decision-maker” to refer to policy and program implementers at senior- or mid-level, as well as individuals who have been involved in the elaboration of policies. Literature exploring women’s involvement in decision-making, and its effect on women-centered policies, suggests that women’s representation can be either *descriptive* or *substantive*. Descriptive representation sees a women decision-maker representing women simply by them being a woman themselves. In such instances, the women decision-maker does not actively push a woman-friendly agenda. Alternately, substantive representation refers to women not only being present in the arena, but actively acting on behalf of women by pursuing women-centered policies that focus on women’s health and health needs [7, 8].

**Fig 1:**
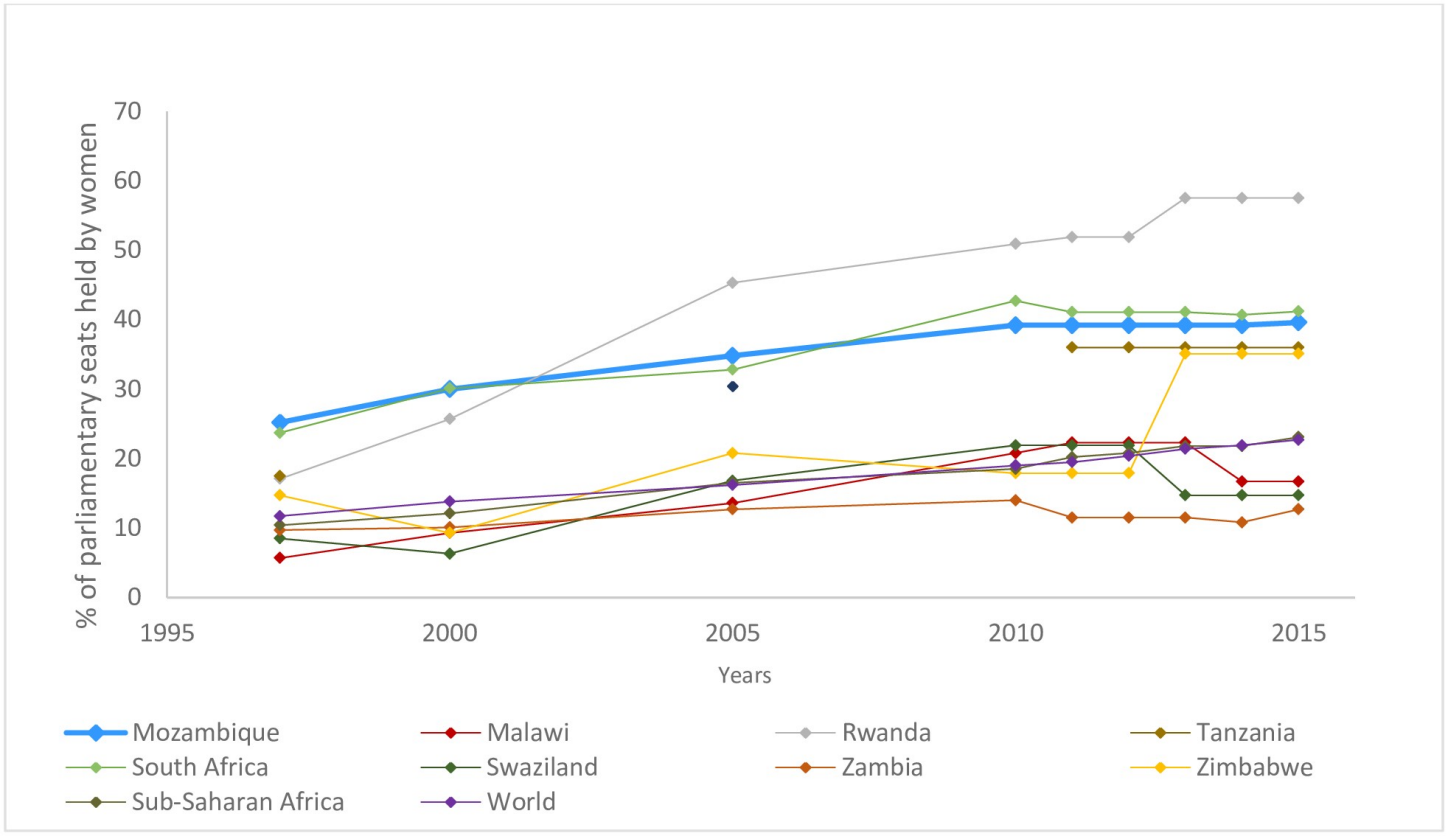
% of parliamentary seats held by women per year in select countries and regions [9].

In Mozambique, women held 39.6% of the seats in the Mozambican parliament in 2015 [9]. This ranks Mozambique as the 40^th^ highest country in the world and 16^th^ in Africa in terms of percentage of parliamentary seats held by women. Fig 1 below shows Mozambique’s standing relative to its neighbouring countries, sub-Saharan Africa and world averages. Mozambique’s ruling party has a quota system for the percentage of women in legislature—originally set at 30%–which was set in 1994 and accomplished in 2000 [10]. Since 1997, Mozambique has sought to promote equal rights for women and men and eliminate all forms of gender discrimination through the review of legal plans, policies and strategies. In addition, Mozambique has a political institutional and legal framework favorable to accounting for and improving gender equality [11].

The Mozambican constitution explicitly prohibits gender-based discrimination in a number of policies and strategic plans. Mozambique has a “National Gender Policy and Strategy for its Implementation”, a “National plan for the Advancement of Women”, and a “Strategic Plan for Gender in the Public Function and for the Prevention and Elimination of Premature Marriages”. The health, energy and mining, education, and agriculture sectors each have a gender-focused strategy. Additionally, laws on topics which disproportionally affect women (such as gender-based violence, family, land, human trafficking, and labour) are also in place [12].

In the health sector, the mission statement of the Strategical Plan for the Health Sector 2014–2019 (PESS), issued by the Mozambican government in 2013, implicitly references women as “vulnerable populations”: “*all Mozambicans*, *especially the most vulnerable groups*, *may benefit from the best health possible*”. This is made explicit in the accompanying text, which states that this mission statement demonstrates the commitment of the health sector to the universality of human rights, “*including the principle of attention to the needs and rights of the most vulnerable groups*, *particularly women*, *children*, *adolescents and youths*”. The PESS’ second principle is “*addressing issues of equity*”, in which addressing gender relations are explicitly identified as priority. The first health priority–“Accelerate progress in reducing maternal and neonatal mortality, including the reduction of the general fertility rate”–is itself a woman-centric priority [13].

Despite these efforts, a 2016 Mozambique gender profile study by the Ministry of Gender, Child and Social Action showed that gender roles, power dynamics and relationships between men and women influence most policy making decisions for health. The study suggests that sociocultural factors continue to result in discrimination against women at the social, political and economic level. While laws exist to protect women’s rights, limited awareness and poor implementation of these laws was highlighted as an ongoing, major challenge. Low social and economic empowerment of women also continue to impact health sector outcomes [12]. This suggests that while women might be significantly represented at the policy-making level, this has not necessarily translated into better health outcomes for women in Mozambique.

In this study, we examine the role of gender norms, roles, and relations in the experiences of female and male senior and mid-level decision-makers in the health sector in Mozambique, including both self-reported policy makers and policy implementers. Our goal was to identify gender-related barriers and facilitators to developing and implementing policies and programs, as perceived by senior and mid-level decision-makers themselves, with the larger aim of identifying opportunities for action to improve women-centered policies.

## Methods

We undertook a qualitative study of mid- and senior-level decision-makers in the health sector in Maputo, Mozambique, from January 2017 to March 2017. The study team was a partnership between the Johns Hopkins School of Public Health (JHSPH) and the Instituto Nacional de Saúde (INS) in Mozambique. Informed written consent was obtained from all participants. Ethical approval was obtained by the Institutional Review Board of Johns Hopkins Bloomberg School of Public Health (IRB#7086) and from the Institutional Committee of Bioethics at INS.

### Sampling

We conducted sampling in two phases: (1) systematic random sampling of an initial sample of 20 individuals; and (2) snowball sampling to identify additional respondents.

From a list of staff of the Mozambique Ministry of Health (MISAU), we identified 95 individuals whose department was deemed relevant to women’s health issues, whose role was deemed relevant to the policy-making process based on job title, and whose position was as senior as Department Chief or higher. Of the 95 identified individuals, 59 were women and 36 were men.

We used systematic random sampling to select a starting sample of 15 women and 5 men. We oversampled female respondents because we were interested in exploring the experiences of female decision-makers in the policy-making process. We used Excel to conduct systematic random sampling, creating two lists of women and men, using the RAND function to order women and men randomly, and selecting every 4^th^ woman and 7^th^ man to further ensure randomization.

At the end of each interview, we asked respondents to suggest three additional participants who they thought would be important to include in our study. Every person that was recommended in this way was added to the sampling list. If the person had not already participated in the study, he or she was contacted by the study team and asked to be interviewed. Such snowball sampling was continued until the study team concluded that saturation had been reached and there was no need to interview more respondents.

### Demographic characteristics

In total, 39 respondents were interviewed, of whom 32 were female, and 7 were male. See Table 1 for additional details on respondents. The majority of respondents were senior level staff with over 5 years of policy-making experience.

**Table 1:** Respondent characteristics.

		Gender	
		Female (n = 32)	Male (n = 7)
**Years of Experience**	0–2	0	1
	3–5	2	0
	5–10	7	2
	10–20	11	3
	20+	11	1
	Unknown	1	0
**Self-identify as policy maker**	Yes	18	2
	No	14	5
**Institutional affiliation**	Ministry of Health	27	5
	NGOs	4	1
	Municipality of Maputo	1	1

### Data collection

We recruited three Mozambican data collectors with experience conducting qualitative research, and held a 2-day training on methodology and to familiarize data collectors with the interview guide.

Data collection took place over a period of 3 months, from January to March, 2017. Data collectors conducted all interviews in Portuguese, at a location and time that was convenient to the respondent. Data collectors scheduled appointments with respondents by phone where possible, or attempted to schedule appointments in-person at the INS when contact information was not available. Data collectors made 3 attempts to reach each respondent, before the respondent was removed from the sampling list and replaced. The next participant on the list was then interviewed until the list was exhausted. After this, participants were selected from the list of individuals recommended by the original participants.

We continued this process until reaching saturation, at 39 participants, when we felt that new themes were no longer being offered or elicited from respondents.

Oral and written consent were obtained from each respondent before the interview was administered. Interviews were recorded using an electronic voice recorder, and data collectors took detailed notes throughout the interview. Data collectors used a semi-structured interview guide developed by the study team, that focused on both the experience of the decision-maker in their respective role, and what they personally felt were priorities around women’s health in Mozambique. Each interview lasted on average between 30 to 45 minutes.

### Data analysis

Data collectors uploaded the interview recordings onto study laptops after each day. Interviews were transcribed verbatim in Portuguese, and data collectors produced short summary reports of each interview. The study team met regularly to discuss emerging themes and to triangulate findings across respondents.

We used an initial review of the literature [14–17] as a point of departure for identifying themes and adjusted as more and different ones emerged from the data through regular debriefing meetings. Transcripts were uploaded into Dedoose (version 7.6.6) and the study team developed a preliminary codebook by conducting a thematic analysis of the data developed from a priori and emerging themes [18]using 8 randomly selected transcripts (20% of total transcripts).

We excluded the initial 8 from the selection and used this first codebook to again code 8 randomly selected transcripts. After making some final adjustments to the final codebook based on our findings in this second round of coding, we then proceeded to code all transcripts in Portuguese line-by-line.

After exploring the data a first time through the coding process, we exported the codes from Dedoose into Excel and re-arranged them by major themes. We used the frequency of occurrence of specific topic as the determinant to define them as major themes. We present a summary of the findings below, and include some excerpts selected because they best represented the major themes.

## Results

Two main topics emerged from the data analysis: (1) perceptions of gender-based discrimination and (2) perceptions of women’s insights on women’s health. Each of these themes represent a different aspect of participants’ gender-related experiences and observations.

### Perceptions of gender-based discrimination

Most women speaking about their own personal experience did not believe that their gender had led to discrimination in the workplace. While no participant argued that gender discrimination does not exist in Mozambique, most reported that this was not an experience that they had personally lived in their work. Some attributed this lack of discrimination to the fact that they work in the health sector, which they perceive to be a woman-friendly field. Others attributed it more to the fact that they are educated women and that their academic or professional qualifications are respected.

“*Me*, *as a woman*, *I never felt discriminated against*. *Maybe it’s because of the field I chose to study*, *but I have not lived an experience of discrimination because I’m a woman*, *no*.”

Woman 25

When mentioning difficulties in the workplace, participants pointed to the structure and limitations of the system, to the lack of data to support their arguments, or to the lack of both human and financial resources to carry out their work. In all cases, participants said that discrimination based on their gender had not been part of that experience.

*“It’s not because I’m a woman*. *It’s because I’m a government worker and I’m leading a team with many shortcomings*. *It’s more because of that*.*”*

Woman 4

Interviewers asked questions specifically about whether the participant’s gender had affected the level of attention or priority given to their work during the development of policies. Female participants did not express feeling that being a woman had affected the attribution of their ideas to others or their upward mobility in the workplace. They did not express that they felt that their work was valued less or more than their male colleagues’.

*“Me*, *personally*, *I can’t complain*. *I don’t think it has any influence*. *I stopped being a director because I wanted to*, *not because someone wanted to remove me and put a man instead*. *No*. *No*, *personally*, *I did not feel this”*

Woman 31

Only one out of the 32 female participants flagged that she felt that sometimes her opinion was challenged more than a man’s would have been. She also mentioned that she had heard comments about women being slow and lazy, which to her had an impact on how women’s opinions and work were perceived. Other participants did speak to women having a higher rate of absenteeism or lateness at work due to competing priorities, but they did not feel that this affected the overall quality of women’s work.

“*It’s a battle*. *You yourself say ‘Hey*! *Listen*!*’*. *If it were someone from the opposite sex who had the same opinion that I have*, *I don’t think it would be challenged this much*. *You almost have to bang on the table*”

Woman 31

Both women and men participants emphasized that their own work is valued independently of their gender. The amount of respect that they receive and their authority in the workplace is seen as equivalent. A person’s personal investment in their work, their character and their technical skills are valued over their gender. The solidity of their arguments, the strength of the evidence behind them and the scientific methodology that they use to build them were also key factors in acceptability, beyond gender.

*“No*, *no*, *no*. *It’s not because I’m a woman*, *but it’s because the arguments I give or we give to convince people are reasonable and true*!*”*

Woman 25

“It doesn’t matter if it’s Johanna […] or John, but it does matter that this person is invested in the topic we’re talking about”

Woman 22

According to participants, this representation of women was not, however, expected to yield any particular results in terms of placing more emphasis on women’s health. Participants expressed feeling that within women and men’s work, respect and influence were deemed generally equivalent and even interchangeable; as a result, they did not see having more women present as either beneficial or detrimental to advancing women’s health.

“The majority of our human resources are female, and I never felt that gender was either an advantage or a disadvantage.”

Woman 29

Finally, according to respondents, women are not only occupying the majority of positions in the health sector, but they are being increasingly nominated to leadership positions, and that leadership positions can be occupied by both men and women.

*“The same positions can be occupied by women and men*. *That’s what’s happening every day*. *This year the director’s a man*, *the next it’s a woman and vice versa”*

Man 6

### Perceptions of women’s insights on women’s health

Despite the consensus among participants that women and men are not treated differently with regards to their work, many mentioned that women may have increased “sensitivity” to topics that she or other female members of her family have experienced. Participants repeatedly used this specific word: sensitivity. From the context, we understood it generally to mean “having firsthand experience of the experience of being a woman and thus having an increased awareness of certain topics”.

According to some participants, being a woman affects personal investment with woman-centered policies, increases sympathy for those affected by the policies, and increases personal satisfaction and pride when there are successful results.

*“I think that as a woman*, *the sensitivity… if we compare men and women*, *women are more sensitive even just for listening*, *for understanding*. *[…] when we want to get somewhere*, *we do*. *When we decide something*, *we fight for it*. *There’s something inherent to women that is special”*

Woman 26

This personal link to women-centered policies by female decision-makers was recognized as something that amplifies the impact of women’s voices and points of view. Women reported personally feeling that this was the case, and some men also supported this feeling.

*“I can understand as a person*, *as a woman*, *as a young person*, *as a friend*, *as a sister*. *I’ve witnessed many cases personally*, *so I think my voice in this technical working group has its impact*.*”*

Woman 11

“I think my voice as a woman in this case is loud and important”

Woman 31

Being able to recognize one’s self in the policies gave some female decision-makers a sense of personal accomplishment and pride in the work being done. Being involved in improving women’s health as women themselves was seen as gratifying on a personal level.

*“I believe that yes*, *[…] we feel fulfilled when we do good for other women*. *It’s the fact that we are women ourselves and we spoke up and shared our experience and we lived these things*, *and now we see things happening*.*”*

Woman 15

It was also suggested that a woman may have better insights into what women-centered policies may or may not be successful, because she has first-hand experience with topics related to being a woman. This sentiment was shared by both men and women.

“You could say ‘injections are the best method’ [for family planning], but a woman might say ‘injections give me convulsions, it doesn’t work for me, I prefer other options, I prefer to monitor my menstrual cycle’ […]. So a woman would be better prepared and might be ready to suggest other options, while for me as a man I would just say ‘No. Injections is the best method’.”

Man 5

However, despite suggesting that being a woman makes them more personally attuned to certain topics, these statements were not linked to any perceived improved or decreased professional performance. Indeed, while women might be seen as more aware of women’s issues, this was not explicitly linked to women being seen at the exclusive authorities on women-centric issues.

*“Maybe when seeing a situation where a woman is involved*, *I might have more empathy since I’m a woman*. *I can identify more with the situation*, *but this is not something that influences work”*

Woman 24

Although respondents saw women as more attuned to women’s issues, the implication was not that men were indifferent to these issues. Both male and female participants describe the men who work in developing or implementing women-centered policies as being actively engaged in women-centric topics. Despite not having personally lived some experiences first-hand, men who contribute to women-centered health policies are seen as empathetic to women-centric causes.

*“We have the department [of Cooperation of the Ministry of Health] itself which has more women*. *It’s a big inequality—there are a lot more women than men*, *but I think even the men who are here are very sensitive to the cause*.*”*

Man 2

## Discussion

Our results suggest three main findings: (1) women who participated in our study generally do not report feeling discrimination in their workplace; (2) decision-makers perceive that women are as well represented in the health sector in Mozambique; and (3) decision-makers perceive women as more personally attuned to women-centric issues than men.

Although at first glance our data suggest that gender bias is not a concern for women in the health sector in Mozambique, contextualizing these findings within existing literature allows us to see other factors at play. Given Mozambique’s longstanding commitment to female representation, it is possible that the effects of having had female representation for so long has indeed normalized female leadership, at least in the health sector. Nevertheless, even in the most resource-rich and gender equality-focused nations, gender discrimination still exists and affects women worldwide [19]. It seems unlikely that Mozambican women would be an exception. In this section, we explore contextual factors that would help explain our findings.

### Considerations of intersectionality

Before discussing these results any further, we want to emphasize that this study only looked at a very specific population group. The participants included in this study were purposefully selected because of their credentials and because of their role as senior decision-makers. The findings from this study must be understood through this lens, where participants come from a place of relative wealth and education. Their perspectives are inherently influenced by these factors since their experience lies at the intersection of wealth, education, and gender. We would strongly caution against expanding the generalizability of these findings across all workplaces in Mozambique, or across all of Mozambican society.

Furthermore, examining these findings through an intersectional lens provides an additional layer to these results. Intersectionality is the notion that a person’s experience is not unidimensional, but rather lies at the intersection of multiple identities. Indeed, each individual belongs to multiple groups relative to their gender, sexual identity, able-bodied status, wealth, class, etc. Some of these are discriminated groups, other are privileged [20]. Quantifying and ranking the exact impact of belonging to each group—discriminated against or not—is essentially unfeasible since a person cannot easily divorce his or herself of parts of their identity.

Female participants in this study generally referenced their positions of power (which in this case implicitly indicates relative wealth), their education, and their organizational and other soft skills as factors affecting their achievements in the workplace. In most cases, they explicitly rejected the notion that their gender had played any role in their professional experience. These responses correlate with Nixon & Humphrey’s findings which suggest that some groups “do not necessarily identify gender oppression as the primary frame through which they understand their lives” [21].

### Female representation in decision-making positions

In Mozambique, women have held at least 30% of parliamentary seats since 2000, exceeding the benchmark of 30% of women in parliament set by the 1995 Beijing Declaration [22]. Another country to achieve this is Rwanda, which has been hailed as an example of equal gender representation in government. In Rwanda, women now hold 61% of parliamentary seats [22]. Rwanda achieved these percentages through a combination of constitutional guarantees, quota systems and electoral structures. However, critics of the use of quota systems—which are also in use in the current elected party in Mozambique—have pointed to public perception of women being appointed to positions of power because they are women, rather than because of their qualifications or professional merit, as being harmful to women’s overall credibility [23]. Nevertheless, prior to the institution of quotas, female representation in government in Rwanda hovered at 25.7% of parliamentary seats [10]. For better or for worse, quotas have allowed female representation to increase much faster than it would presumably have without them.

A notable difference between Mozambique and Rwanda is that while the currently elected political party in Rwanda does have additional incentives to ensure a strong female representation, the Rwandese constitution itself has a 30% quota for positions held by women [23]. In contrast, Mozambique’s currently elected party—the *Frente de Libertação de Moçambique* (FRELIMO)–has a quota system in place for women representation “in all party structures at all levels and in all lists for any election” originally set at 30%, but now at 40% of positions [24]. The Mozambican constitution, however, does not [25]. Other Mozambican political parties do not have explicit gender-sensitive policies in place to guarantee or explicitly promote a more gender-balanced representation. While most declare that gender equality is a priority or at least a consideration, the institutional mechanisms that guarantee female representation are lacking [24, 26]. However, women in Mozambique have a lower literacy rate than men, have fewer educational opportunities, and are culturally socialized to not be outspoken. These elements contribute to reducing the pool of qualified potential women in positions of power [12]. Even if the quota system isn’t adopted by other parties, without some concrete plan on how to ensure female representation is maintained or without integrating something of the kind in the constitution itself, the advances that Mozambique has made in terms of female representation may become vulnerable.

While what we have thus far discussed focuses on politics directly, Ministers and Deputy-Ministers are appointed—and dismissed—by the president directly, and their political affiliation tends to be a factor in being selected or not [24, 25]. The Ministry of Health is not a party structure *per se*, but its leadership aligns with the political party that appointed it, and which does support having a minimum of 40% of women at all levels. With women representing 54% of the staff in the Ministry of Health and women present at all decision-making levels within this institution—including the Minister of Health herself, Mozambique has met the objective of achieving significant levels of female representation in the health sector—at least quantitatively.

### Descriptive or substantive?

Extensive literature exists on the notion of the “critical mass”, defined as the percentage of representation where a group will go from figurehead to having significant impact [27–29]. Minority groups—women in the decision-making sphere, in our case—when represented in low proportions can have some impact merely by their presence. They have an experience which has been shaped by their experience of being socialized as women, and have insights and perspective that those who have not, do not. Nevertheless, the fear of backlash from the male majority for being too outspoken might keep some women from expressing their own views because they are aware that their gender causes them to be under heightened scrutiny. Some might even overcompensate by adopting even less gender-sensitive positions than their male colleagues. Furthermore, the token woman may be asked to speak on behalf of all women, without acknowledging the vast differences in experiences that individuals who identify as women face [28]. Nevertheless, even low representation does bring a female voice to the table, albeit an imperfect and perhaps perfunctory one.

The critical mass theory proposes that once a minority group reaches a certain proportion, the group will shift from the descriptive representation described above to a substantive one. With enough members present, individuals’ voices are amplified and the group can express viewpoints that are no longer quite as unilateral. Per this theory, eventually the general culture will shift to better accommodate the minority group. 30% is often pointed to as the proportion where this shift happens, and is the target set in the 1995 Beijing Declaration. The Mozambican government, with 39.6% of parliamentary seats held by women, has exceeded this percentage. More specifically for our study, 54% of positions in the Ministry of Health are held by women [30].

Mozambique is nearing 25 years of having the 30% quota system in place. From our interviews—and the available quantitative data—it would seem that Mozambique has moved past descriptive representation and into substantive. Our participants speak of being a woman as an advantage when working on women-centric health issues. They state that their status as a woman gives their voice more weight to speak on these issues. They insist that they do not think they are or have been silenced, dismissed or excessively criticized because of their gender. Participants also speak of male and female leadership being viewed as essentially interchangeable. All of these elements do suggest that at least to some extent, women are well enough represented that they are no longer tokens. It is possible that having sustained this level of female representation—both in government and in the health sector—for such an extended period has affected the way female representation is perceived.

### Going beyond the critical mass

Childs and Krook (2008) propose going beyond the critical mass theory of simply increasing the numbers of women, and focusing on critical actors of any gender instead. Indeed, the grossly simplified subtext behind focusing strictly on percentages of women in politics is that all women are feminists and will pursue feminist agendas, and all men are not and will not. This thought process—elements of which were reflected in our findings—is problematic for the following reasons.

Firstly, it shifts the responsibility of advocating for women-centered policies from all actors to strictly women. Even if women were to have a more personal investment in advancing women’s issues, women’s rights are human rights. The responsibility of ensuring that they are respected and upheld lies on society as a whole. Throughout the analysis of our findings, the notion of women having increased “sensitivity” towards women-centric issues was repeated by both women and men, from more junior to very senior members. This expected “intrinsic sensitivity” is also reflected in the literature, with findings showing that women are expected to be more altruistic than men by both women and men [31] and on average do behave more altruistically than men [32]. This apparently widespread perception is often used as the basis for justifying why pursuing gender equality is “a woman’s thing”, because it is perceived that women somehow innately know what is best for women and are therefore the ones who should speak on behalf of women.

Secondly, it removes men from the equation as the important actors for gender equality that they can be. Representation matters, but so does the concerted efforts of allies who, while not part of the group that they advocate for, can use their positions of privilege to bring more attention to disadvantaged groups. Indeed, our interviews with participants showed that there is a perception that the men who work in public health are “sensitive to the cause”, and that they are just as invested as women in achieving better health for all. This finding—men being perceived as equally invested in women’s health as women—is in agreement with going beyond the critical mass theory. Assuming that all men are not interested in gender equality reinforces the trope that gender issues are women’s issues instead of acknowledging that gender equality benefits societies as a whole—men included.

Thirdly, not all women necessarily see gender issues as a priority. Indeed, female participants in this study expressed that they did not think that they had lived experiences of gender-based discrimination in their workplace. Because they do not see gender discrimination as an issue in their own experiences, they might not necessarily prioritize measures to minimize its impact. This may have been a result of their own intersectional positions in society, in which any gender discrimination that may have been present was mitigated by their privileged economic and educational background. This also supports the idea that a woman or man may not necessarily be in a position to represent all women or men, particularly those who may be marginalized as a result of their intersecting identities. Our findings support the idea that women may not necessarily always act for women, which once again supports going beyond critical mass theory.

Moving the question from “is this parliamentary seat held by a woman or by a man?” to “is this parliamentary seat held by an individual who systematically prioritizes gender equality considerations in their political behaviour or not?” would lead to a more relevant discussion in Mozambique and elsewhere. Nevertheless, we acknowledge that measuring this indicator in a standardized way even within the same country—never mind across countries and cultures—would be a herculean task. Given how much definitions for gender identities, gender roles and gendered expectations vary within and between countries, this would be extremely difficult.

### Limitations

Our use of snowball sampling as the second stage of our sampling strategy may have introduced bias, as it is possible that participants might have been more likely to refer subsequent participants who were likeminded. However, we believe that the bias potentially introduced here is largely offset by our additional use of systematic random sampling, increasing the likelihood that our initial pool of candidates had a variety of opinions and experiences, and thus that subsequent participant selection would have resulted in a variety of experiences as well.

While we made a conscientious effort to probe on people’s experience of gender beyond simply asking whether they had felt or witnessed discrimination or not, our study tool was by no means exhaustive. We did not explicitly ask if participants had experienced sexual harassment, had equal access to educational and training opportunities, or had adequate family support. These are issues that are linked to individuals’ gendered experiences in the workplace, and including them in our interview guide may have led to a different outcome. We would suggest using a gender framework to explore some of the themes further in future studies.

Implying that gender discrimination may have impacted an individual’s career—either positively or negatively—detracts from the meritocratic narrative of one’s position having been gained strictly through one’s own efforts. Acknowledging this may cause emotional discomfort or distress. Cognitive dissonance may occur when information or experiences that jar against a deeply held belief are subconsciously ignored or dismissed [33]. Minimizing or rationalizing the impact of gender discrimination is a way that women have dealt with the discomfort or pain of recognizing that it may have affected their experiences. Attributing discriminatory experiences as being the natural order of things or to cultural norms may also occur [34, 35]. We acknowledge that data presented in this paper come from participants’ perceptions of their experiences and thus has been filtered through their interpretative lenses.

## Conclusion

Mozambique is unique in that it has had at least 30% of parliamentary positions held by women for nearly 20 years; significantly longer than many other African countries. This success in terms of female representation seems to be reflected in our study participants’ own personal experiences. While we recognize the limitations of our study, and the important factors of intersectionality and descriptive or substantive roles, we found little to suggest that gender discrimination is a problem professionally for female senior- and mid-level decision-makers in the health sector in Mozambique.

However, while the current level of female representation should be celebrated, it does not negate the need for continued or greater focus on gender equality. Sustained action, and efforts from critical actors of all genders, are still needed for promoting gender equality, and for improving women’s health throughout the country. Encouragingly, our results suggest that female and male participants do seem to be engaged with women’s health, and that the sustained representation of women in government may also have elevated women-centric health policies. More research is needed to examine participants’ perceptions in more depth to determine how their responses and experience were shaped by their gender.

## Supporting information

S1 File Interview guide in English. Interviewer guide used by data collectors, in English.
(DOCX)

S2 File Interview guide in Portuguese. Interviewer guide used by data collectors, in Portuguese.
(DOCX)

S3 File Recruitment and consent in English. Recruitment and consent forms used by data collectors, in English.
(DOCX)

S4 File Recruitment and consent in Portuguese. Recruitment and consent forms used by data collectors, in Portuguese.
(PDF)
