# Supplementary material for: Reaching substantive female representation among decision-makers: A qualitative research study of gender-related experiences from the health sector in Mozambique
Source: PLoS One. 2018 Nov 15;13(11):e0207225. doi: 10.1371/journal.pone.0207225 (PMC6237335; doi:10.1371/journal.pone.0207225)
Supplement: S1 File — Interviewer guide used by data collectors, in English. (DOCX) [file pone.0207225.s001.docx]

**Guião Auxiliar**

*Experiences of female and male policy makers in Mozambique in developing and implementing progressive policies for women’s health*

| **Data:**  **Entrevistador ID (Iniciais):**  **Hora de Inicio:**  **Hora de Fim da Entrevista:**  **Código do Respondente:**  **Profissão/Cargo:**  **Idade:**  **Sexo:** |
| --- |

**Demographical questions:**

1. How long have you been working in this position? (time in service)
2. Can you describe your main tasks and responsabilities?
3. During the time where you have worked in your current position, have you had any contact with the design and implementation of policies related to women’s health?
4. Can you give one or more examples of these policies, and explain your role in their design and implementation?

**Part 1: Perception of women’s health problems**

1. In your perception, what aspects do you consider to be relevant for women’s health in Mozambique? Why?

*Probe: Intervention area, (sexual health, maternal...), specific health services, specific illnesses..*

*Examples to give:*

- *Do you think most women would agree with you (if so, why or why not)? Do you think most men would agree with you (if so, why or why not)?*

1. Of the aforementioned aspects, which ones do you consider receive the most attention from the decion-makers/policy-makers in the last 2-3 years? Why?
2. How are these aspects reflected in women’s health policies (how were these aspects incorporated/prioritized?)?

*Probe: How do these aspects appear in this policy? Is it in the plans?*

1. Is there any other key policy on women’s health that has been developped in the last 2-3 years?

*Probe: What factors could lead to this policy being proritized over other ones? (emergencies, prevalence, financing, etc...)*

1. Beyond what has already been mentioned, do you think that there are other aspects of women’s health that deserve more attention?
2. Why do you consider that these issues did not receive much attention? What do you think has influenced this?

**Parte 2: Experiences in the decision-making process**

1. What has been your experience in the process of developing these policies?
2. What was your contribution? Could you give me a specific example?

*Further examples to give:*

- *Do you think your contribution would have been different if you were of the other gender?*
- *Do you think the valorization of your contribution would have been different if you were of the other gender?*
- *If the inverviewee says he/she make does not make the final decisions:*
  - *Who –according to you – has the final say in making policy decisions? Do you think his/her gender influences that?*
  - *Approximately how many final decision-makers are male compared to how many are female? Why do you think that is?*

1. During your participation or contribution to this proces, did you encounter some difficulties? (in which policy, specifically). Can you give some examples of the difficulties that you’ve had?

*Probe: Positive aspects/facilities that you had in the development of policies (work conflicts, lack of time, overlapping activities- on the professional level; personal-home, lack of attention/fincancing/academic level/category/changes in leadership/lack of consideration/respect)*

*Further examples to give:*

- *Have you ever felt like your voice was not heard? Or that your ideas were dismissed?*
- *Have you been talked over during meetings when you tried to express an idea or opinion?*
- *Have you been excluded from participating in meetings?*
- *Have your ideas or thoughts been taken by others?*

1. Do you consider that someone of the opposite gender would have had the same difficulties in this process? Why?

*Examples to give:*

- *For example, do you think that someone of the opposite gender would receive more respect during meetings or when speaking?*
- *Do you think that you need to prove your ability more than someone of the opposite gender?*

1. Of the policies designed or implemented in which you have participated, were there any in which your contribution exceeded your expectations?
2. What factors (internal or external) contributed to the level of your involment in with the design/implementation of these policies?

*(Probe: Experiences or personal experiences, family environment, gender, )*

1. Do you consider that the fact that you are a woman/man has some influence on your level of attention or priority given during the development of policies on:
   1. Women’s health? *Probe: Why? How so?*
   2. Men’s health? *Probe: Why? How so?*
2. Is there anything about policies and experiences with health policies that has not been discussed today and that you’d like to share with me?

| PI Name | Timothy Roberton, International Health |
| --- | --- |
| Study Title | Experiences of female and male policy makers in Mozambique in developing and implementing policies for women’s health |
| PI Version No./Date | Version 3.3/January 18^th^ 2017 |
