# Supplementary material for: Reaching substantive female representation among decision-makers: A qualitative research study of gender-related experiences from the health sector in Mozambique
Source: PLoS One. 2018 Nov 15;13(11):e0207225. doi: 10.1371/journal.pone.0207225 (PMC6237335; doi:10.1371/journal.pone.0207225)
Supplement: S2 File — Interviewer guide used by data collectors, in Portuguese. (DOCX) [file pone.0207225.s002.docx]

**Guião Auxiliar**

*Experiences of female and male policy makers in Mozambique in developing and implementing progressive policies for women’s health*

| **Data:**  **Entrevistador ID (Iniciais):**  **Hora de Inicio:**  **Hora de Fim da Entrevista:**  **Código do Respondente:**  **Profissão/Cargo:**  **Idade:**  **Sexo:** |
| --- |

**Perguntas Demográficas:**

1. A quanto tempo trabalha nesta posição? *(tempo de serviço)*
2. Pode descrever as suas principais tarefas e responsabilidades?
3. Durante o tempo de trabalho na sua posição actual, teve algum contacto com o desenho e implementação de políticas relacionadas com a saúde da mulher?
4. Pode dar um ou mais exemplo dessas políticas, e explicar o seu papel para o desenho e implementação das mesmas?

**Parte 1: Percepção sobre os problemas de saúde das mulheres**

1. Na sua percepção, que aspectos considera relevantes na saúde da mulher em Moçambique? Porque?

*Probe: Area de intervencao (Saude sexual...materna....) , servicos de saude especificos, doencas em particular...*

1. Dos aspectos mencionados anteriormente, quais considera que tiveram mais atenção por parte dos decisores/fazedores politicos nos últimos 2-3 anos? Porque?
2. Como é que estes aspectos estão reflectidos nas políticas sobre a saúde da mulher (Como é que estes aspectos que foram incorporados/priorizados?)?

*Probe: Como estes aspectos aparecem nessa politica? Estao dentro dos planos?*

1. Existe alguma outra política chave sobre a saúde da mulher que tenha sido desenvolvida nos últimos 2-3 anos?

*Probe: Que factores podem levar a que se priorizem umas politicas no lugar de outras?*

*(emergencias, prevalencia, financiamento, etc..)*

1. Para além do já mencionado, acha que existem outros aspectos da saúde da mulher que deveriam merecer mais atenção?
2. Por que considera que estes assuntos não receberam muita atenção? O que terá influenciado?

**Parte 2: Experiências no processo de elaboração de políticas?**

1. Qual tem sido a sua experiencia no processo de elaboração destas políticas?
2. Qual foi a sua contribuição? Pode dar um exemplo especifico?
3. Durante a sua participação ou contribuição neste processo, enfrentou alguma dificuldade? ( Em que politicas, especificamente) Pode citar com exemplos que tipo de dificuldades teve?

*Probe: Aspectos positivos/ facilidades que teve na elaboração de politicas*

*(conflictos laborais, falta de tempo, sobreposicao de actividades- a nivel professional;pessoal-casa, falta de atencao/ interesse/financiamento/nivel academico/categoria/ mudanca de lider/ falta de consideracao/respeito)*

1. Considera que alguem do sexo oposto teria as mesmas dificuldades neste processo? Porque?
2. Das politicas desenhadas ou implementadas em que participou, houve alguma na qual o o seu contributo superou as suas expectativas? Por que?
3. Que factores (internos ou externos) contribuiram para o nivel do seu envolvimento no desenho/ implementação dessas políticas?

*(Probe: Experiencias ou vivencias pessoais, entorno familiar, genero, )*

1. Considera que o facto de ser (homem ou mulher) tem alguma influencia no seu nivel de atencao ou prioridade dada durante a elaboração de políticas sobre :
   1. A saude da mulher? *Probe:* *Por que? De que forma?*
   2. A saude do homem? *Probe: Por que? De que forma?*
2. Existe alguma coisa sobre as políticas e experiências de saúde que não tenha sido comentadas hoje, que gostaria de partilhar comigo?

| PI Name | Timothy Roberton, International Health |
| --- | --- |
| Study Title | Experiences of female and male policy makers in Mozambique in developing and implementing policies for women’s health |
| PI Version No./Date | Version 3.3/Janury 18^th^ 2017 |
