# Supplementary material for: Reaching substantive female representation among decision-makers: A qualitative research study of gender-related experiences from the health sector in Mozambique
Source: PLoS One. 2018 Nov 15;13(11):e0207225. doi: 10.1371/journal.pone.0207225 (PMC6237335; doi:10.1371/journal.pone.0207225)
Supplement: S3 File — Recruitment and consent forms used by data collectors, in English. (DOCX) [file pone.0207225.s003.docx]

**All Participants**

**PI Name: Timothy Roberton**

**Study Title: Experiences of female and male policy makers in Mozambique in developing and implementing policies for women’s health**

**IRB No.: 00007086**

**PI Version/Date: Version 1.2/March 31^st^, 2016**

Thank you for your time in hearing about our study. We are working to understand the role and experiences of female and male policy makers within the government and the development of women-centric health programs and policies. We ask you to join our work because of your experiences as a health policy maker in Mozambique. You do not have to join, it is your choice, but your participation might help future clients.

If you say yes, we will ask you to answer a number of questions on your experiences in being a part of the policy-making process, as well as your thoughts on the current state of women’s health in Mozambique. It will take approximately 60 to 90 minutes.

You may be uncomfortable answering some questions that we ask you. You do not have to answer all the questions and you may stop at any time.

You will not experience a direct benefit from answering these questions. We will use the answers to help inform our understanding of the current role of both female and male policy makers within government, and to understand the current state of women’s health in Mozambique.

We will not let anyone outside our work see your answers and health information. We will do our best to keep your information safe by not writing down your name. When we share your information with other researchers, we will ask them to use the same protections.

We will not pay you to help us.

The interview will be recorded, and this recording will be reviewed after the interview is over by the data collector and possibly other study staff. Identifying information will not be asked of you, however in the case that identifying information is included in the recording, it will not be included in the notes. The recording will be destroyed upon completion of the data analysis by study staff.

Do you have any questions? You may contact Katia Ngale (+258 8273737360, kngale1@jhu.edu) about your questions or problems with this work. You may contact the Ethics Committee (Insert name and number here) which approved this study about any problems or concerns.

Do I have your permission to record this interview?

May I begin?
