# Supplementary material for: Reaching substantive female representation among decision-makers: A qualitative research study of gender-related experiences from the health sector in Mozambique
Source: PLoS One. 2018 Nov 15;13(11):e0207225. doi: 10.1371/journal.pone.0207225 (PMC6237335; doi:10.1371/journal.pone.0207225)
Supplement: S4 File — Recruitment and consent forms used by data collectors, in Portuguese. (PDF) [file pone.0207225.s004.pdf]

Guião de Recrutamento

Todos Participantes

Experiências de decisores políticos do sexo feminino e masculino em Moçambique no desenvolvimento e implementação de políticas de saúde da mulher

Bom dia/Boa tarde, o meu nome é _____________ e sou membro do grupo de estudo da Plataforma Nacional de Avaliação, uma iniciativa conjunta do Instituto Nacional de Saúde e da Johns Hopkins University. Nós estamos conduzindo um estudo relacionado com decisores políticos do sexo feminino e masculino em Moçambique e as suas experiências no processo de elaboração de políticas. Nos gostaríamos de entrevistar mulheres e homens decisores políticos que têm tomado parte no desenvolvimento e implementação de políticas de saúde, especialmente relacionadas com a saúde das mulheres. O objetivo desde estudo é identificar os obstáculos e fatores facilitadores que afetam os decisores políticos do sexo feminino e masculino como participantes do processo de decisão política, e como isso pode afetar o desenvolvimento de programas de saúde da mulher.

Nós gostaríamos de falar com o/a Sr./Sra. para aprender sobre suas experiências em fazer parte desse processo, e para compreender as suas percepções sobre a saúde das mulheres em Moçambique.

Se disser que sim, pediremos que responda algumas perguntas sobre a sua experiência como parte no processo de desenho de políticas, e também a tua opinião sobre o actual estado de saúde das mulheres em Moçambique. A nossa conversa levará aproximadamente 60 a 90 minutos.

Há riscos mínimos de participar neste estudo: (a) Você pode se sentir desconfortável em falar de alguns aspectos ligados as actividades por si desenvolvidas. Estas podem incluir perguntas sobre a sua experiencia no processo de criação de políticas. Por isso poderá também sentir-se pouco a vontade para responder algumas perguntas relacionadas ao seu trabalho. Não é obrigado(a) a responder a todas as perguntas e poderá parar a entrevista a qualquer momento.

Não deixaremos que ninguém fora da nossa equipa de trabalho veja as suas respostas. Daremos o nosso melhor para manter a tua informação em segurança e não registaremos o seu nome. Quando partilharmos a tua informação com outros investigadores, pediremos que usem as mesmas proteções.

O Sr(a) não terá nenhum benefício directo ao responder as perguntas. No entanto usaremos as tuas respostas para ajudar-nos na compreensão do actual papel dos decisores políticos governamentais, e compreender o actual estado de saúde das mulheres em Moçambique. Não receberá pagamento pela sua ajuda.

As informações que você e outros participantes da pesquisa irão fornecer serão arquivadas no INS, e de forma segura serão partilhadas com os membros da equipe de estudo a Johns Hopkins University, que é a instituição parceira do INS neste estudo.

Todas as informações que você vai dar são confidenciais e serão mantidas em segurança para proteger a privacidade dos seus argumentos.

Se permitir participar no estudo, está entrevista será gravada, e esta gravação será revista pelo entrevistador e possivelmente por outros membros da equipe do estudo. Informações sobre sua identificação não serão perguntadas, no entanto, no caso em que alguma informação de identificação esteja incluída nas notas, a gravação será destruída após a conclusão da análise dos dados pela equipe do estudo.

Se você concordar em participar deste estudo, por favor assinar o termo de consentimento. Obrigado por participar neste estudo.

ID do Entrevistador (iniciais): ____

Assinatura:___________________________________ Data: ___________________

<u>DECLARAÇÃO DO PARTICIPANTE</u>

Fui informado verbalmente e por escrito sobre este estudo e compreendo de quê se trata. Sei também a quem contactar se necessitar mais informação. Compreendo que a confidencialidade será mantida. Compreendo que sou livre de sair do estudo ou retirar o participante para o qual sou o representante legal em qualquer altura, sem que isto afecte os cuidados normalmente recebidos.

Nome do participante no estudo :____

Assinatura:___________________________________ Data: ___________________

Nome do PI	Timothy Roberton, International Health
Titulo do estudo	Experiências de decisores políticos do sexo feminino e masculino em Moçambique no desenvolvimento e implementação de políticas de saúde da mulher
IRB No.	
Versão No./Data	Versão 1.4/ 26 de Agosto de 2016
